# Effects of Glycerol Fatty Acid Esters on Growth Performance, Methane Emissions, and Rumen Microbial Flora of Dabieshan Beef Cattle

**DOI:** 10.3390/vetsci13010092

**Published:** 2026-01-16

**Authors:** Junjie Nie, Xinye Li, Yongchang Luo, Hongxian Li, Yong Zhu, Chao Chen, Jinling Hua

**Affiliations:** 1 College of Animal Science, Anhui Science and Technology University, Chuzhou 239000, China; junjie6263@163.com (J.N.); 14792810387@163.com (X.L.); luoyongchang2023@163.com (Y.L.); lihongxian20221@outlook.com (H.L.); 2 Taihu Jiuhong Agricultural Comprehensive Development, Anqing 246400, China; zhuyong1172@163.com

**Keywords:** Dabieshan beef cattle, meat quality, rumen microbiota, serum biochemical parameters

## Abstract

Glycerol fatty acid esters (GFAEs) exhibit potential applications in ruminant production, including enhancing animal performance, improving nutrient utilization, and modulating rumen function. However, studies on indigenous Dabieshan beef cattle are lacking. This study aimed to evaluate GFAE’s effects on their performance, meat quality, and rumen function. Thirty 2-year-old cattle (294.73 ± 3.21 kg; mean ± SD), were randomly divided into three groups (*n* = 10): on a dry matter (DM) basis, CON (basal diet), 0.05 GFAE (basal + 0.05% GFAE), 0.1 GFAE (basal + 0.1% GFAE), fed for 60 days. The crude protein (CP) digestibility of the 0.05 GFAE group showed a significant 4.55% increase compared with the CON group, while that of the 0.1 GFAE group was significantly elevated by 2.76% relative to the CON group. For key meat quality indices of Dabieshan beef cattle, compared with the CON group, the 0.05 GFAE and 0.1 GFAE groups showed increases in L value by 10.14% and 7.11%, respectively (*p* = 0.042); decreases in shear force by 5.24% and 1.48%, respectively (*p* = 0.024); and increases in ether extract(EE) content by 10.91% and 2.33%, respectively (*p* = 0.019). Compared with the CON group, the 0.05 GFAE and 0.1 GFAE groups showed significant alterations in key serum biochemical indices of Dabieshan beef cattle: TP (total protein) levels elevated significantly by 6.44% and 13.04%, respectively (*p* = 0.010); total antioxidant capacity (T-AOC) increased significantly by 33.96% and 46.23%, respectively (*p* = 0.001); UREA concentrations decreased significantly, by 22.67% and 33.53%, respectively (*p* = 0.002); superoxide dismutase (SOD) activity rose significantly, by 7.30% and 7.99%, respectively (*p* = 0.020); and malondialdehyde (MDA) content declined significantly, by 20.25% and 28.03%, respectively (*p* = 0.040). Relative to the CON group, dietary supplementation with GFAE significantly increased ruminal butyrate concentrations, with the 0.05 GFAE and 0.1 GFAE supplemented groups exhibiting respective increments of 17.38% and 18.03% (*p* = 0.025). Both Groups 0.05 GFAE and 0.1 GFAE reduced CH4 emissions (*p* = 0.005) and elevated *Prevotella* abundance (*p* = 0.001). The study findings revealed that dietary supplementation with GFAE at concentrations of 0.05% and 0.1% of dry matter resulted in substantial decreases in daily methane emissions, representing respective reductions of 6.91% and 11.63% compared to the control group (*p* = 0.005). At the species level of the rumen microbial community, the relative abundance of the genus *Prevotella*_sp. was significantly elevated by 60.52% and 38.48% in the 0.05 GFAE and 0.1 GFAE groups, respectively, when contrasted with the CON group (*p* = 0.001). Collectively, these results demonstrate that the inclusion of dietary 0.05% GFAE supplementation conferred multifaceted benefits to Dabieshan beef cattle, thereby highlighting its potential as a viable strategy to enhance the sustainability of beef cattle production systems.

## Simple Summary

In the context of modern ruminant production, where enhancing animal performance, health, and product quality while minimizing environmental impact is paramount, Glycerol fatty acid esters (GFAE), a class of natural lipid-derived compounds with diverse biological activities, have garnered increasing attention in the field of animal nutrition as promising functional feed supplements. This growing interest arises from their intrinsic emulsifying and antimicrobial attributes, along with their capacity to promote growth performance, improve nutrient utilization efficiency, and regulate rumen microbial fermentation processes. The central objective of this study is therefore to investigate the efficacy of GFAEs as a novel feed additive to improve rumen function and overall animal performance. Employing a completely randomized experimental design, thirty 24-month-old Dabieshan beef cattle were allocated randomly into three experimental groups, with 10 individuals assigned to each group: control (CON, basal diet), group 0.05 GFAE (basal + 0.05% GFAE of dietary dry matter), and group 0.1 GFAE (basal + 0.1% GFAE of dietary dry matter). Results: groups 0.05 GFAE and 0.1 GFAE showed higher apparent crude protein (CP) digestibility; group 0.05 GFAE had superior meat quality by brighter color, higher fat content, and tender texture. Both groups, 0.05 GFAE and 0.1 GFAE, had increased ruminal butyrate, reduced serum harmful substances, enhanced antioxidant capacity, and elevated *Prevotella*_sp. abundance, as well as decreased daily CH_4_ emissions. This study integrated growth performance, meat quality, serum metabolism, rumen fermentation, methane emissions, and rumen microbiota to evaluate the regulatory effects of GFAE on the productivity and sustainability of Dabieshan beef cattle, aiming to provide a theoretical basis for optimizing their nutritional management.

## 1. Introduction

Genetic analyses have identified two primary groups within Chinese native cattle: southern breeds with ancestry tracing back to Asian Bos indicus and northern breeds with roots in Eurasian Bos taurus [1], which Southern Chinese cattle exhibit distinct phenotypic traits and adapt well to local environmental conditions [2]. Dabieshan beef cattle are an indigenous Chinese breed primarily raised for beef production. They tolerate extreme weather and unprocessed feed and exhibit strong disease resistance. Recognized as a prominent local breed, Dabieshan beef cattle are listed in the National Catalog of Chinese Livestock and Poultry Genetic Resources and mainly distributed in the Dabieshan of Anhui Province, a region that gives the breed its name, derived from the crossbreeding of Bos taurus and Bos indicus, Dabieshan beef cattle exhibit rich genetic diversity [3]. Long-term outdoor grazing and selective breeding for draft purposes have shaped Dabieshan beef cattle with distinct morphological traits, including a compact stature, robust bone structure, well-proportioned build, and strong limbs, alongside enhanced disease resistance [4]. Previous studies have shown that Dabieshan beef cattle exhibit higher crude fat content, lower shear force values, and more favorable meat quality traits—including reduced cooking loss highlighting their high potential for high-quality beef production [5]. However, most existing studies on GFAE application in ruminants have been confined to commercial breeds [6], and there is a complete lack of targeted research on indigenous breeds represented by Dabieshan beef cattle. Notably, Dabieshan beef cattle indicus hybrid with a unique rumen microbial community shaped by their genetic background and local grazing environment [3]; this distinct microbial structure implies that their physiological and metabolic responses to GFAE may diverge significantly from those of commercial breeds. This critical knowledge gap not only hinders the understanding of GFAE’s regulatory mechanism in indigenous ruminants but also restricts the development of breed-specific nutritional strategies tailored to local beef cattle resources. GFAE are a class of ester compounds formed via the esterification of glycerol with fatty acids, encompassing short-chain fatty acid esters (SCFAE), medium-chain fatty acid esters (MCFAE), and long-chain fatty acid esters (LCFAE) [7]. Due to their unique molecular structure and physiological functions, GFAEs have garnered growing attention in animal production. Improved feed conversion efficiency to reduce production costs, and enhanced animal health through the regulation of gastrointestinal microbiota [8,9,10]. Research has demonstrated that fatty acids improve feed conversion ratio (FCR) in broilers [11], enhance nutrient digestibility [12], immune function, and antioxidant capacity in weaned piglets [13], increase feed intake and average daily gain (ADG) in calves [14], boost antioxidant capacity [15] and furthermore, mitigate enteric methane emissions in ruminant production [16]. Building on the established roles of GFAE as critical metabolites derived from intestinal microbiota, these compounds have been shown to markedly enhance feed utilization efficiency, modulate gastrointestinal microbial community structure, and sustain metabolic equilibrium in the host, thereby synergistically boosting animal growth performance, immune competence, and antioxidant activity. Building on these well-documented effects, we hypothesized that incorporating GFAE into the diet of Dabieshan beef cattle would improve feed conversion ratios, concurrently promote growth performance and antioxidant capacity, mitigate enteric methane production, and refine meat quality parameters. This study, therefore, aimed to empirically validate this hypothesis and establish a theoretical framework for developing nutritional strategies tailored to the specific requirements of indigenous cattle breeds.

## 2. Materials and Methods

### 2.1. Research Location and Materials

The feeding trial was conducted at Jiuhong Beef Cattle Farm, Baili Town, Taihu County, Anhui Province, China (115°57′36.58″ E longitude, 30°40′5.21″ N latitude; WGS-84 coordinate system). Glycerol fatty acid esters (GFAE, MF20241008) were obtained from Guangzhou Baishi Biotechnology Co., Ltd., Guangzhou, China, as a light grayish-brown powder or granular product. According to the manufacturer’s specifications, its main components included medium- and short-chain fatty acids (25–50% of total weight), postbiotics (primarily metabolites of lactic acid bacteria, 40–50%), and feed-grade silica (10%).

### 2.2. Experimental Design

Thirty 24-month-old indigenous Dabieshan beef cattle (initial body weight: 294.73 ± 3.21 kg, mean ± SD) were randomly divided into 3 groups (*n* = 10/group), housed and fed individually throughout the trial. The control (CON) group received an unsupplemented basal diet (formulated to meet NY/T 815-2004 [17] beef cattle nutritional standards), while the 0.05 GFAE and 0.1 GFAE groups were supplemented with GFAE at 0.05% and 0.1% of dry matter intake (DMI), respectively. The trial comprised a 7-day adaptation period (to acclimate animals to diets/housing) followed by a 60-day experimental phase; dietary composition and nutrient levels are detailed in Table 1.

### 2.3. Growth Performance

Before the commencement of the formal trial, all cattle underwent a 12 h overnight fast and were individually weighed to establish their initial body weight (IBW). Final body weight (FBW) was recorded on day 60 of the trial, and average daily gain (ADG) was calculated using the formula: (FBW − IBW)/duration of the trial. During the entire trial period, daily feed offered and refused were recorded individually for each animal to determine individual dry matter intake (DMI). Feed conversion ratio (FCR) was subsequently calculated as the ratio of DMI to ADG, which reflects the amount of dry matter consumed per unit of body weight gain. Growth performance parameters were determined using the following equations:

- Average daily dry matter intake (ADMI) = total dry matter intake/trial duration (t)
- Average daily gain (ADG) = (FBW − IBW)/trial duration (t)
- Feed Conversion Ratio (FCR) = average daily feed intake (ADFI)/average daily gain (ADG)

### 2.4. Nutritional Analysis

Crude protein content was analyzed in accordance with GB/T 6432-2018 [18]; dry matter levels were quantified via GB/T 6435-2014 [19]; ash content was measured following GB/T 6438-2007 [20]; calcium concentration was determined per GB/T 6436-2018 [21]; crude lipid content was evaluated based on GB/T 6433-2025 [22]; and phosphorus content was analyzed pursuant to GB/T 6437-2018 [23]. For dietary fiber fractionation, Acid Detergent Fiber (ADF) and Neutral Detergent Fiber (NDF) were quantified using the Van Soest fiber analysis protocol [24]; this approach entails sequential treatment of samples with neutral detergent solution to separate NDF, followed by acidic detergent solution to isolate ADF, thereby separating the distinct fiber fractions present in the samples.

### 2.5. Apparent Total Digestibility

During the 50th day of the formal trial, a 7 day digestion and metabolism experiment was initiated, encompassing a 3-day adaptation phase followed by a 4-day collection period for feces and urine [25]. Five animals were randomly selected from each group, and digestion and metabolism parameters were evaluated using the total excreta collection method [26]. These selected cattle were housed individually (one per pen) to enable the separate collection of feces and urine. Feces and urine were collected before daily feeding; freshly collected feces were immediately treated with 10% sulfuric acid (Zhonghui Wanxin Co., Ltd., Chuzhou, China) to prevent ammonia nitrogen volatilization. All feed and fecal samples were dried in an oven at 65 °C for 48 h; subsequent to drying, the samples were ground to pass through a 1 mm sieve and stored in airtight containers until laboratory analysis. Dry matter was determined using GB/T 6435-2014, crude protein using GB/T 6432-2018, crude fat using GB/T 6433-2025, ash using GB/T 6438-2007, calcium using GB/T 6436-2018, and phosphorus was determined according to GB/T 6437-2018. The concentrations of ADF and NDF are quantified using the method described by Van Soest et al. [24]. The formula used to calculate the apparent digestibility of nutrients is presented below:

Apparent digestibility (%) = [(nutrient intake − nutrient excretion in feces)/nutrient intake] × 100%.

### 2.6. Meat Quality

At the conclusion of the formal trial, all experimental cattle underwent a 24 -h fast prior to humane slaughter. Following exsanguination, the longissimus thoracis muscle located between the 12th and 13th thoracic vertebrae was excised, and its cross-sectional area was quantified using sulfuric acid-impregnated graph paper (Model MX01, Meixing Co., Ltd., Jiaxing, China). Approximately 500 g of longissimus dorsi (LD) muscle sections (2 cm thick) were collected for subsequent meat quality evaluations. Colorimetric parameters (L* for lightness, a* for redness, b* for yellowness) of the LD muscle were determined using a CR-400 chromameter (Konica Minolta, Tokyo, Japan), with values averaged from three distinct measurement locations. For shear force determination, LD muscle samples were heated in a water bath maintained at 80 °C until the internal temperature reached 70 °C. After cooling to ambient temperature, shear force was measured using an RH-N50 texture analyzer (Guangdong Lianfang Biotechnology Co., Ltd., Panyu District, Guangzhou, China). To assess cooking loss, LD muscle samples were trimmed into 2 cm × 2 cm × 3 cm cubes with all visible connective tissue removed, weighed, and sealed in impermeable plastic bags. Samples were then heated in a water bath for 30 min, cooled to room temperature for 15 min, blotted to remove surface moisture, and reweighed to calculate cooking loss percentage.

Cooking loss was calculated using the formula:

Cooking loss (%) = [(Pre-heating weight − Post-heating weight)/Pre-heating weight] × 100%

For water holding capacity (WHC) determination: Meat samples were sandwiched between 16 layers of filter paper (8 layers on each side) and placed on an RH-100 instrument (Guangdong Lianfang Biotechnology Co., Ltd., Panyu District, Guangzhou, China). A pressure of 35 kg was applied for 5 min, and the sample was weighed immediately after pressing.

WHC was calculated as:

WHC (%) = [(Weight before pressing − Weight after pressing)/Weight before pressing] × 100%

Ether extract (EE) and crude protein (CP) contents in LD muscle samples were determined following the method reported by Yang et al. [6].

Meat specimens were sandwiched between 16 layers of filter paper (8 layers above and 8 layers below) and secured in an RH-100 press instrument. A 35 kg pressure was applied for 5 min; immediately after pressing, the specimen was reweighed to determine WHC. EE and CP concentrations in muscle samples were determined following the protocol established by Yang et al. [6].

### 2.7. Serum Biochemical Parameters

On the 60th day of the formal trial, all experimental cattle were slaughtered. This procedure had been reviewed and approved by Anhui Institute of Science and Technology’s Institutional Animal Care and Use Committee (IACUC), and conducted per humane, animal welfare-aligned beef cattle slaughter standards to minimize animal stress and suffering. On the same day, 10 mL of blood was drawn from the tail vein of the beef cattle on an empty stomach. The blood was left to stand at room temperature for 30 min, then centrifuged (Allegra X-30R, Beckman Coulter, Inc., Brea, CA, USA) at 3000× *g* for 10 min. The serum was collected and divided into 1.5 mL sterile centrifuge tubes (PCR-2-C, AXYGEN, Union City, CA, USA) and stored at −20 °C (MD-25L98, Midea, Guangzhou, Guangdong, China) and stored at −20 °C (MD-25L98, Midea, Guangzhou, Guangdong, China). The following serum biochemical parameters were quantified via enzyme-linked immunosorbent assay (ELISA; MeiKang Biotechnology Co., Ltd, Qingdao, Shandong, China.): catalase (CAT; ml092621), glutathione peroxidase (GSH-PX; ml076447), malondialdehyde (MDA; ml094963), superoxide dismutase (SOD; ml076328), total antioxidant capacity (T-AOC; ml076332), albumin (ALB; H103), total protein (TP; H102), urea (UREA; H106W), total cholesterol (TC; H202), triglycerides (TG; H201), high-density lipoprotein cholesterol (HDL-C; H203T), and low-density lipoprotein cholesterol (LDL-C; H207). All detections were performed using a Hitachi 3110 (Hitachi 3110 automatic biochemical analyzer, Hitachi High-Technologies Corporation, Tokyo, Japan) automatic biochemical analyzer.

### 2.8. Methane, Carbon Dioxide Parameters

Methane (CH_4_) and carbon dioxide (CO_2_) emissions from Dabieshan beef cattle were measured using an automated head-chamber (AHC) system [27]. Prior to formal measurements, the cattle were acclimated to the AHC system for 7 days, and the formal monitoring lasted for 16 consecutive days. The experiment was conducted in two phases, with CH_4_ emission data collected over 4 consecutive days per phase. The specific sampling times were as follows: on the 1st sampling day, measurements were taken at 05:00, 13:00, and 21:00; on the 2nd sampling day, at 07:00, 15:00, and 23:00; on the 3rd sampling day, at 09:00, 17:00, and 01:00 (next day); and on the 4th sampling day, at 11:00, 19:00, and 03:00 (next day). The second phase started immediately after the first phase, with the measurement procedure repeated.

Airflow rate was calibrated and recorded using a vortex flowmeter (Jinbeici Instrument Co., Ltd., Heping District, Shenyang, China). Gas samples were extracted at 2 L/min through the air collection pipe downstream of the fan, routed through a 2.0 µm air filter, and analyzed every 1 s using a portable Greenhouse Gas Analyzer (GGA-30p, Los Gatos Research, Los Gatos, CA, USA). This measurement method was adapted from the protocol developed by Wang et al. [27].

### 2.9. Rumen Fermentation Characteristics

On day 60 of the experiment, 10 biological replicates (experimental cattle) were selected for each experimental group. Rumen fluid was collected orally, using a rumen fluid collection tube (GCYQ-1/A, Greede, Shanghai, China) following a 24 h fast, and subsequently filtered through four layers of sterile gauze. The pH value was immediately measured using a portable pH meter (S220-K, Mettler Toledo, Greifensee Switzerland). The filtered rumen fluid was stored at −80 °C (DL-86, Zhongke Xileng Co., Ltd., Ningbo, Zhejiang, China) for subsequent analysis. Rumen NH_3_-N concentration was determined via the phenol-hypochlorite method [28]. Volatile fatty acids (VFA) were measured using a gas chromatograph (A91Plus, Changzhou Pano Instrument Co., Ltd., Changzhou, China) according to the protocol described by Ran et al. [29].

### 2.10. Gastric Microbial Community Analysis

Fifteen frozen rumen fluid samples (5 biological replicates per experimental group) were submitted to Anhui General Biology Technology Co., Ltd. (Anqing, China) for metagenomic sequencing analysis. The detailed experimental procedures are outlined as follows: Extraction and quality assessment of genomic DNA (gDNA); Fragmentation of gDNA into smaller segments; End repair of DNA fragments to generate blunt ends, followed by the addition of a 3′-terminal adenine (A) overhang to convert them into sticky ends; Ligation of DNA adapters containing index sequences to both ends of the sticky ends via base complementarity; Size selection using magnetic beads to collect target fragments within a predefined length range; PCR amplification to append indexes to the terminals of target fragments, followed by the completion and quality verification of sequencing libraries; Immobilization of sequencing libraries onto the sequencing chip through bridge PCR; On-instrument sequencing using either the Illumina HiSeq or MiSeq platform, with the platform selection determined by the fragment size, all sample handling and sequencing procedures were performed in strict accordance with the detailed operational protocols outlined in Supplementary Method S1 [6,30].

### 2.11. Statistics and Analysis

Statistical analyses of growth performance, nutrient digestibility, serum biochemical indices, methane emissions, and rumen fermentation parameters were conducted using SPSS 25.0 software. Before conducting formal statistical assessments, data normality and homoscedasticity were verified through the Shapiro–Wilk test and Levene’s test. Data satisfying these criteria were analyzed via one-way analysis of variance (ANOVA) to quantify the regulatory effects of dietary GFAE supplementation. When significant disparities were identified (*p* < 0.05), subsequent pairwise comparisons were performed using Duncan’s multiple range test. Statistical significance was set at *p* < 0.05, with distinct superscript letters in result tables denoting statistically significant intergroup differences. Additionally, Pearson correlation analysis was carried out in R software (version 4.3.1) to explore associations between the relative abundance of dominant rumen bacterial taxa and rumen fermentation indices in GFAE-supplemented cattle.

## 3. Results

### 3.1. Growth Performance and Apparent Nutrient Digestibility

Compared with the CON group, no statistically significant differences were detected in FBW, TBW, ADG, DMI, and F/G between the 0.05 GFAE and 0.1 GFAE supplemented groups (*p* > 0.05; Table 2). With respect to apparent nutrient digestibility in Dabieshan beef cattle, GFAE supplementation exhibited a tendency to increase DM digestibility (*p* = 0.058), though this effect did not reach statistical significance. Apparent CP digestibility was significantly elevated in both the 0.05 GFAE and 0.1 GFAE groups relative to the CON group (*p* = 0.002), corresponding to increases of 4.55% and 2.76%, respectively. No significant variation in EE, NDF, and ADF digestibility was observed across all experimental groups (*p* > 0.05; Table 3).

### 3.2. Meat Quality Characteristics

To assess the effect of GFAE on meat quality traits of Dabieshan beef cattle, comprehensive trait analysis was employed; it was employed in this study to conduct a comprehensive assessment of the associated meat quality traits. The findings demonstrated that GFAE supplementation significantly enhances the L* value and EE content of beef while decreasing its shear force. Specifically, relative to the CON group, the L* value was elevated by 10.14% in the group 0.05 GFAE and 7.11% in the group 0.1 GFAE (*p* = 0.042); meanwhile, the shear force in the groups 0.05 GFAE and 0.1 GFAE was significantly reduced by 5.24% and 1.48% compared with the CON group (*p* = 0.024). Additionally, the EE content in the group 0.05 GFAE was significantly increased by 10.91% (*p* = 0.019). No significant differences were observed in cooking loss, a* and b* value, WHC, or eye muscle area among the three experimental groups (*p* > 0.05) (Table 4).

### 3.3. Determination of Serum Biochemical and Antioxidant Indices

For the purpose of evaluating the effects of GFAE on serum biochemical profiles in Dabieshan beef cattle, serum parameters were determined via enzyme-linked immunosorbent assay (ELISA) kits and an automated biochemical analyzer, and the results indicated that relative to the CON group, serum TP levels were significantly elevated by 6.44% and 13.04% in the 0.05 GFAE and 0.1 GFAE supplemented groups, respectively (*p* = 0.010), UREA concentrations were markedly decreased by 22.67% and 33.53% in the 0.05 GFAE and 0.1 GFAE groups, respectively (*p* = 0.002), T-AOC increased significantly by 33.96% (0.05 GFAE group) and 46.23% (0.1 GFAE group) (*p* = 0.001), SOD activity was notably enhanced in both GFAE-supplemented groups (by 7.30% and 7.99%, respectively; *p* = 0.020), serum MDA content declined significantly by 20.25% (0.05 GFAE group) and 28.03% (0.1 GFAE group) (*p* = 0.040), while no statistically significant variations were observed in serum concentrations of ALB, TC, TG, HDL-C, LDL-C, GSH-PX, or CAT among all treatment groups (all *p* > 0.05; Table 5).

### 3.4. Rumen Fermentation Parameters

Relative to the CON group, dietary supplementation with GFAE significantly increased ruminal butyrate concentrations, with the 0.05 GFAE and 0.1 GFAE supplemented groups exhibiting respective increments of 17.38% and 18.03% (*p* = 0.025). In contrast, statistically significant variations were not observed among all treatment groups with respect to ruminal ammonia nitrogen (NH_3_-N), pH, Acetate, Propionate, Butyrate, Isobutyric acid, Isovaleric acid, Valeric acid, TVFA, and A/P (all *p* > 0.05; Table 6).

### 3.5. Methane (CH_4_) and Carbon Dioxide(CO_2_) Emissions

The results demonstrated that the levels of CH_4_ emissions in groups 0.05 GFAE and 0.1 GFAE were significantly lower than those in the CON group (*p* = 0.005). No significant differences were observed in CO_2_ emissions across all experimental groups (Table 7).

### 3.6. Analysis of Alpha Diversity Index of Rumen Microorganisms

**Figure 1:**
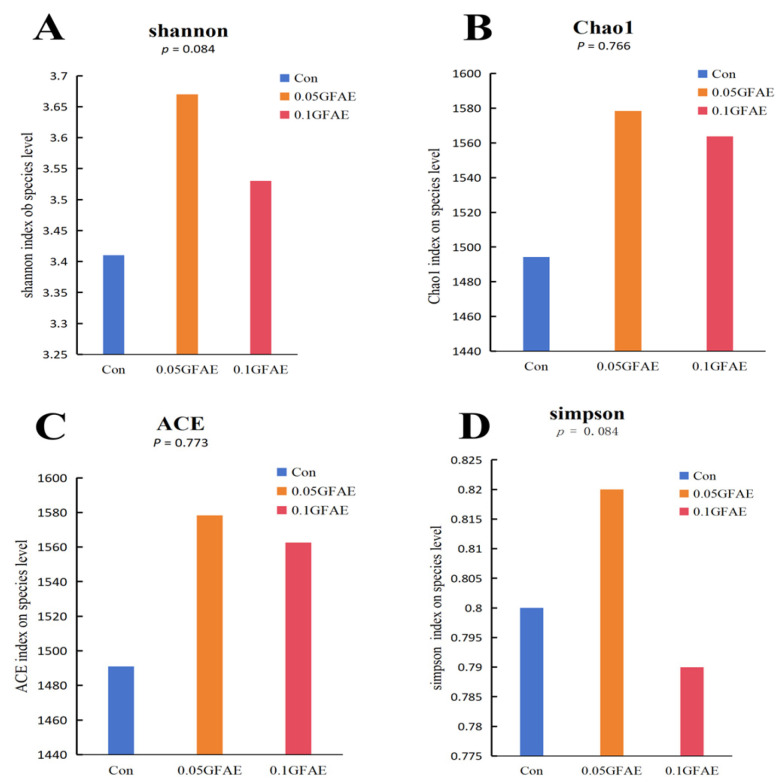
Analysis of the alpha diversity index of rumen microorganisms. (**A**): Shannon index; (**B**): Chao1 index; (**C**): ACE (Abundance-based Coverage Estimator) index; (**D**): Simpson index.

Alpha diversity characterizes the richness and evenness of microbial species, with the Chao1 and ACE indices employed to quantify species richness, and the Shannon and Simpson indices used to gauge species evenness. Following 60 days of dietary GFAE supplementation, the α-diversity of ruminal microbiota among all experimental groups was evaluated via these four indices (Figure 1). The results demonstrated that in comparison with the CON group, the 0.05 GFAE supplemented group exhibited a noticeable upward tendency in both the Shannon (*p* = 0.084) and Simpson (*p* = 0.084) diversity indices; nevertheless, these observed variations did not reach statistical significance.

### 3.7. Microbial Community Comparison

**Figure 2:**
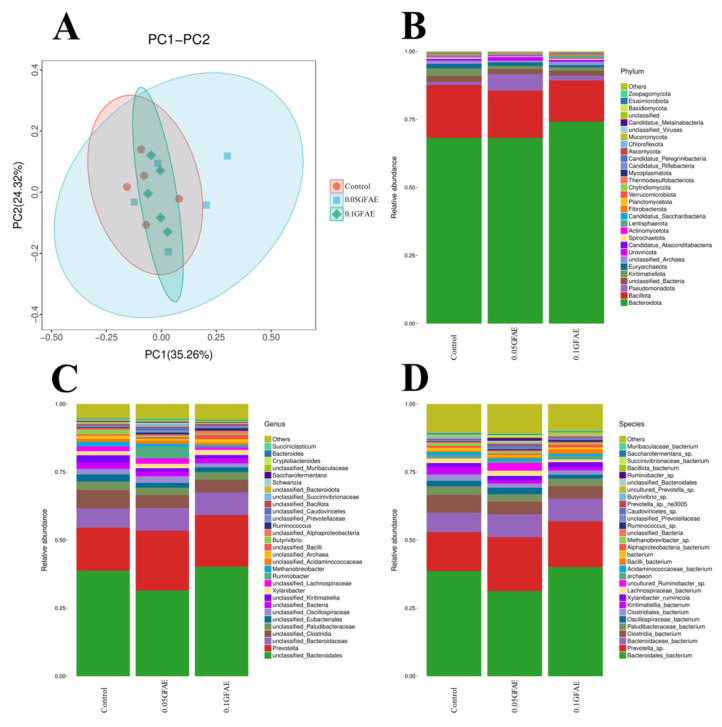
Effects of glycerol fatty acid esters supplementation on rumen microbiota in Dabieshan beef cattle. (**A**): Principal Coordinate Analysis (PCoA, based on Bray–Curtis dissimilarity) and Analysis of Similarities (ANOSIM); (**B**): Rumen microbial community composition at the phylum level; (**C**): Rumen microbial community composition at the genus level; (**D**): Rumen microbial community composition at the species level. The ordinate values (1.00, 0.75, etc.) in panels (**B**–**D**) represent the relative abundance (decimal form) of microbial taxa.

β-diversity assessment was performed for ruminal fluid specimens of cattle in each group via PCoA, as illustrated in Figure 2A, revealing intergroup overlap across the three cohorts. No marked distinction was detected in rumen microbial community compositions among the experimental groups. The primary principal component (PC1) explained 35.26% of the total variance, while the secondary principal component (PC2) accounted for 24.32%. These findings demonstrate that the three groups exhibit no substantial variations in microbial structural makeup, indicating that dietary supplementation with GFAE does not alter the rumen microbial composition of beef cattle.

Predominant bacterial phyla consisted of *Bacteroidota* and Proteobacteria, with Pseudomonas (a genus in the *Proteobacteria phylum*) identified as one of the prevalent genera across all groups; *Bacteroidota* accounted for ~70% of the total microbial population. Compared with the CON group, the 0.1 GFAE experimental group showed an 8% increase in *Bacteroidota*’s relative abundance (Figure 2B). At the species level, the top three dominant bacterial taxa were *Bacteroidales* bacterium, *Prevotella*, and *Bacteroidaceae* (Figure 2D). At the species level of the rumen microbial community, the relative abundance of the genus *Prevotella*_sp. was significantly elevated by 60.52% and 38.48% in the 0.05 GFAE and 0.1 GFAE groups, respectively, when contrasted with the CON group (*p* = 0.001, Appendix A Table A1).

### 3.8. Associations Between Ruminal Fermentation Indicators and Microbial Community Profiles

**Figure 3:**
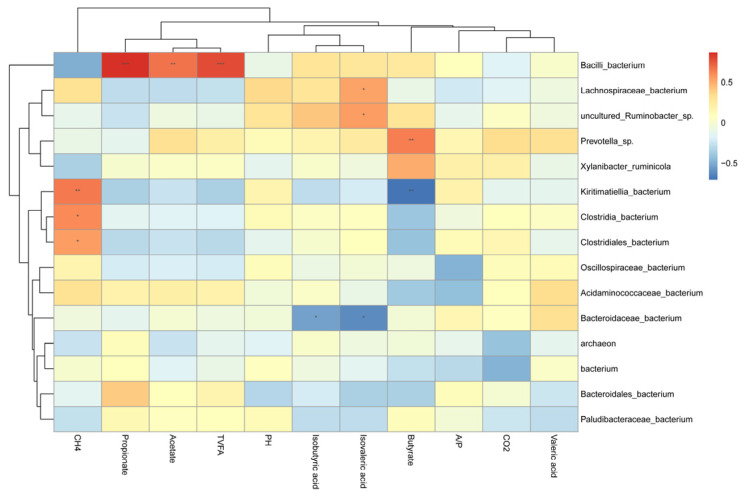
Correlation between rumen microbial communities with rumen fermentation characteristics and gas emission. CH_4_: Methane; CO_2_: Carbon Dioxide; A/P (acetate/propionate ratio); Butyrate (butyric acid); Acetate (acetic acid); TVFA (Total volatile fatty acid); Propionate (propionic acid); This heatmap illustrates Pearson correlation coefficients between the top 15 genera and fermentation parameters: red blocks indicate positive correlations, while blue blocks indicate negative correlations. Significance levels are denoted as follows: * *p* < 0.05; ** *p* < 0.01; *** *p* < 0.001.

Pearson correlation assay was conducted to investigate the relationships between the relative abundance of major ruminal bacterial taxa and ruminal fermentation indicators in beef cattle receiving GFAE fortified diets. Notably, CH_4_ displayed a highly significant negative association with *Kiritimatiella*_bacterium, *Clostridia*_bacterium, and *Clostridiales*_bacterium (*p* < 0.01). Moreover, ruminal propionate, acetate, and TVFA were strongly positively correlated with *Bacilli*_bacterium (*p* < 0.01). Furthermore, ruminal isobutyric acid and isovaleric acid demonstrated a substantial negative association with *Bacteroidaceae*_bacterium (*p* < 0.05). Meanwhile, butyrate showed a highly significant positive relationship with *Prevotella*_sp (*p* < 0.01), as presented in Figure 3.

## 4. Discussion

The present study observed that supplementing finishing diets with two levels of GFAE did not elicit significant changes in the growth performance of Dabieshan beef cattle. Notably, a point to note regarding this work is that only two supplementation levels were evaluated (rather than a gradient of increasing doses), so our findings do not fully capture potential dose-dependent responses to GFAE in this breed. Subsequent research incorporating multiple graded GFAE levels would help to further clarify the relationship between supplementation dosage and growth performance in Dabieshan beef cattle. Masmeijer et al. observed no marked impacts of glycerol esters of saturated short and medium-chain fatty acids on growth variables in veal calves [31]. GFAE exerted only a modest regulatory effect on productive traits [32]. Dietary supplementation with 1.5% crude corn oil, a fatty acid source, has no significant effects on the growth performance of finishing beef cattle, consistent with the limited effects of GFAE on Dabieshan beef cattle in this study [33].

Increased CP digestibility comprehensively enhances the marbling, tenderness, juiciness, and nutritional value of beef by promoting protein synthesis, optimizing fat metabolism, and enhancing antioxidant capacity [34]. Supplementation of GFAE enhanced CP digestibility in the current study, consistent with the observations of Almudena Cabezas et al. [34]. DM digestibility tended to increase quadratically with GFAE supplementation. Low-dose GFAE supplementation may enhance nutrient absorption through improved emulsifying activity, yet the extra absorbed energy is not efficiently directed toward tissue accretion likely due to unchanged energy expenditure or protein deposition pathways, as observed with butyrate derivatives in dairy calves [14,35]. Excessive GFAE, conversely, could suppress intake [36]. Moreover, high GFAE doses may lower ruminal pH, compromising cellulolytic bacterial activity and reducing VFA production [16]. In the study, ruminal pH was maintained within the optimal range with no reduction in DMI, indicating that the tested concentrations did not reach the over-supplementation threshold. However, it should be noted that we cannot definitively conclude whether concentrations beyond the scope of this study would trigger the over-supplementation threshold, which represents a limitation of our research. Luan et al. [16] reported improved DM digestibility without significant ADG changes when medium-chain fatty acids were added to cattle diets, corroborating our findings. Future work should integrate whole-animal energy-balance measurements with protein-turnover techniques to define optimal inclusion levels [28,29].

In the present trial, GFAE supplementation significantly elevated longissimus muscle L* values and EE content, while reducing shear force. Carvalho et al. [37] attributed increased L* to higher intramuscular fat, whose optical properties enhance light reflectance consistent with our observations. GFAE also upregulates lipogenic genes (FASN, PPAR-γ) [38,39] promoting lipid deposition in muscle, disrupting muscle-fiber continuity, and reducing shear force, which aligns with these mechanisms. Serum biochemical indices reliably reflect nutritional metabolism and redox homeostasis in ruminants [40]. Consistent with this mechanism, Guo et al. [41] further confirmed in bovine mammary epithelial cells that butyrate, a key metabolite affected by GFAE supplementation in our study, activates the Nrf2 pathway by promoting Nrf2 nuclear accumulation and H3K9/14 acetylation via GPR109A, directly enhancing antioxidant capacity and alleviating oxidative damage. In this study, GFAE supplementation significantly elevated TP, T-AOC and SOD activity, while lowering MDA and concentrations, indicating enhanced systemic antioxidant defense, this aligns with the Keap1-Nrf2-ARE pathway. Kensler et al. [42] systematically elaborated the core mechanism of this pathway, where disruption of Keap1-Cullin3 interaction liberates Nrf2 for nuclear translocation and ARE binding, thereby up-regulating antioxidant enzyme transcription. This pathway-related antioxidant phenotype is validated in ruminants [43]. Murayama et al. [44] reported that medium- and short-chain fatty acids increase serum SOD activity while reducing MDA in calves—consistent with our results. SOD, ubiquitous in aerobic organisms, catalyzes the dismutation of superoxide radicals (O_2_^−^) into H_2_O_2_ and O_2_, mitigating oxidative damage to cellular membranes, proteins, and DNA [45]. MDA, a lipid peroxidation marker, declined markedly in GFAE-fed animals, reflecting attenuated oxidative injury [46]. Luan et al. [16] similarly observed that medium-chain fatty acids reduce MDA and modulate ruminal microbiota to promote butyrate-producing bacteria, increasing ruminal butyrate concentrations.

After consuming carbohydrates and dietary protein, ruminal fermentation produces VFAs, which are the main energy sources for ruminants and their microbiota. The current study observed that GFAE significantly increased butyrate levels, supported by Salinas-Chavira et al. [47]. Butyrate regulates innate immunity in ruminants by stimulating bovine neutrophils and potentiating platelet-activating factor activity via free fatty acid receptor 2-mediated Ca^2+^ influx and MAPK phosphorylation [48]. Additionally, GFAE-induced butyrate elevation may correlate with reduced CH_4_ production [49], prompting subsequent CH_4_ emission measurements.

GFAEs have gained increasing attention as a promising nutritional strategy for mitigating enteric CH_4_ emissions in ruminants [50]. Building on this background, the present study employed GFAE supplementation to reduce CH_4_ output in Dabieshan beef cattle, with no concomitant changes in CO_2_ emissions observed. MCFAs are known to suppress ruminal carbohydrate fermentation and exert inhibitory effects on methanogenic archaea and ciliate protozoa, thereby down-regulating the hydrogenotrophic pathway of CH_4_ biosynthesis [51]. Notably, both MCFAs and LCFAs have been consistently shown to exhibit anti-methanogenic potential in both in vitro and in vivo models [52]. This is supported by Luan et al. [16], who demonstrated that MCFAs supplementation in batch culture significantly reduced CH_4_ production—consistent with the decreased CH_4_ emission levels observed in our current study. However, the exact regulatory mechanisms underlying this GFAE-mediated CH4-suppressing effect remain to be fully verified and elucidated. In terms of rumen microbial communities, *Kiritimatiella* belongs to the phylum *Verrucomicrobia*, and studies have demonstrated that members of this phylum can indirectly inhibit methane production by competing for methanogenic precursors such as hydrogen [53]. A large number of hydrogenotrophic bacteria, which reduce methanogenic precursors by consuming H_2_ [54]. Studies have demonstrated that Firmicutes act as key producers of VFAs in the rumen, directly generating acetate, propionate, and other TVFA through carbohydrate degradation. The abundance of Firmicutes exhibits a significant positive correlation with propionate, acetate, and TVFA, which is directly linked to their functional role in carbohydrate metabolism [55].

In community ecology, alpha diversity reflects species richness and evenness within a community, while beta diversity reflects inter-community compositional differences [56]. Results indicated that GFAE supplementation did not significantly affect ruminal microbiota alpha or beta diversity. At the phylum level, GFAE increased *Bacteroidota* abundance. As a dominant ruminal phylum, *Bacteroidota* degrades cellulose, hemicellulose, and proteins and adapts rapidly to nutrient changes, producing succinate, acetate, and butyrate to supply host energy [57,58,59]. At the genus level, GFAE increased *Prevotella*_sp. abundance, consistent with Petri et al. [60]. Kong et al. [61] found reduced *Prevotella* in the rumen of ketotic dairy cows, with fat supplementation restoring microbial balance. At the species level, GFAE increased *Prevotella* and *Bacteroidaceae* bacterium abundance while reducing *Methanobrevibacter* sp. *Bacteroides thetaiotaomicron*, a *Bacteroidaceae* member, degrades starch and yeast mannan via glycoside hydrolases in the starch utilization system, producing oligosaccharides, acetate, and succinate via the succinate pathway [62,63]. Oligosaccharides released by *B. thetaiotaomicron* are utilized by butyrate-producing bacteria, whose butyrate promotes tight junction protein expression in intestinal epithelial cells, enhancing barrier function [64,65]. For methanogenesis regulation, saturated fatty acids inhibit methanogenesis by increasing methanogen membrane permeability and reducing *Methanobrevibacter* survival [66]. Roskam et al. [67] reported that polyunsaturated fatty acid-rich oils reduce CH_4_ emissions in dairy cows, which is consistent with the results observed in the present study that CH_4_ emissions were decreased in beef cattle fed with GFAE; however, the specific regulatory mechanisms underlying this effect remain to be elucidated.

## 5. Conclusions

The study examined the effects of 0.05% and 0.1% GFAE supplementation on Dabieshan beef cattle. The results demonstrated that GFAE significantly improved crude protein digestibility, increased meat lightness, elevated ether extract content, and reduced shear force. Additionally, GFAE actively modulated serum biochemical parameters, leading to increased total protein levels and antioxidant capacity, as well as decreased urea and malondialdehyde concentrations. Furthermore, GFAE supplementation reduced methane emissions, increased butyrate levels in rumen fluid, and elevated the relative abundance of *Prevotella*_sp. In conclusion, the preliminary evidence suggests that the 0.05 dosage may be preferable. However, the current study was limited to only two supplementation levels, which prevents a comprehensive assessment of potential dose-dependent risks or benefits. Therefore, future research should involve larger-scale studies with multiple dosage gradients to validate these effects, determine the optimal supplementation range, and elucidate the underlying mechanisms of action.

## Figures and Tables

**Table 1:** Nutrient profile and ingredient composition of the control diet (as-fed basis).

Ingredients	Percentage (%)
Straw	44.28
Corn	11.63
Wheat bran	6.17
Baijiu Distillers grains	36.77
Nacl	0.65
Premix ^a^	0.50
Total	100.00
Nutrient levels ^b^	
NEg (Mcal/kg)	1.24
DM	50.01
CP	12.73
EE	3.84
NDF	37.66
ADF	28.13
Ash	12.79
Ca	0.61
P	0.33

DM: dry matter; CP: crude protein; EE: ether extract; NDF: neutral detergent fiber; ADF: acid detergent fiber; ASH: Crude ash; Ca: Calcium; P: Phosphorus; ^a^: The premix provided the following per kilogram of the basal ration: vitamin A, 3000 IU; vitamin D3, 450 IU; tocopherol acetate, 200 mg; Fe, 90 mg; Zn, 8 mg; Mn, 40 mg; Cu, 20 mg; I, 0.5 mg; Se, 0.5 mg; Co, 0.2 mg; ^b^: All nutrient levels except DM are expressed on a dry matter basis. NEg is defined as net energy for gain. The NEg values were established following the guidelines of NY/T 815.

**Table 2:** Influence of glycerol fatty acid esters on the growth performance of Dabieshan beef cattle.

Items	CON	0.05GFAE	0.1GFAE	SEM	*p*-Value
IBW (kg)	297.07	292.42	294.71	3.21	0.854
FBW (kg)	324.57	325.71	322.70	3.16	0.922
TBW (kg)	27.50	33.29	27.99	1.42	0.188
ADG (kg/d)	0.45	0.55	0.46	0.02	0.187
DMI (kg/d)	5.60	5.96	5.55	0.16	0.614
F/G	12.75	10.82	12.18	0.52	0.501

CON: basal diet; 0.05 GFAE: basal + 0.05% GFAE of dietary dry matter; 0.1 GFAE: basal + 0.1% GFAE of dietary dry matter. IBW: initial body weight; FBW: final body weight; TBW: Total body weight gain; ADG: average daily gain; DMI: dry matter intake; F/G: DMI/ADG. SEM: Standard Error of the Mean.

**Table 3:** Effect of glycerol fatty acid esters on the Apparent Nutrient Digestibility of Dabieshan cattle.

Items	CON	0.05GFAE	0.1GFAE	SEM	*p*-Value
DM (%)	65.71	67.52	66.36	0.32	0.058
CP (%)	61.34 ^b^	64.13 ^a^	63.03 ^a^	0.40	0.002
EE (%)	64.07	64.18	67.04	3.69	0.793
NDF (%)	55.58	56.49	55.98	3.96	0.993
ADF (%)	45.97	49.24	48.01	2.54	0.089

CON: basal diet; 0.05 GFAE: basal + 0.05% GFAE of dietary dry matter; 0.1 GFAE: basal + 0.1% GFAE of dietary dry matter. DM: dry matter; CP: crude protein; EE: ether extract; NDF: neutral detergent fiber; ADF: acid detergent fiber. SEM: Standard Error of the Mean; Distinct superscript letters (a, b) in the same row denote statistically significant intergroup differences (*p* < 0.05); values with the same or no superscript letters indicate no significant difference (*p* > 0.05).

**Table 4:** Effect of glycerol fatty acid esters on the Quality of Dabieshan beef cattle.

Items	CON	0.05GFAE	0.1GFAE	SEM	*p*-Value
L*	30.09 ^b^	3.14 ^a^	32.23 ^ab^	0.51	0.042
a*	19.36	19.77	21.07	0.74	0.616
b*	5.87	6.20	6.50	0.39	0.808
cooking loss (%)	30.80	27.34	27.94	2.23	0.800
WHC (%)	27.00	23.56	24.79	0.95	0.390
Shear force (N)	37.23 ^b^	35.28 ^a^	36.68 ^b^	0.33	0.024
EMA (cm^2^)	33.11	33.42	34.47	0.68	0.730
EE (%)	12.01 ^b^	13.32 ^a^	12.29 ^b^	0.19	0.019
CP (%)	36.94	41.00	35.10	2.36	0.680

CON: basal diet; 0.05 GFAE: basal + 0.05% GFAE of dietary dry matter; 0.1 GFAE: basal + 0.1% GFAE of dietary dry matter. L*: Lightness; a*: Red-Green Chromaticity Coordinate; b*: Blue-Yellow Chromaticity Coordinate; WHC: Water holding capacity, EMA: Eye muscle area; EE: ether extract; CP: crude protein. SEM: Standard Error of the Mean; Distinct superscript letters (a, b) in the same row denote statistically significant intergroup differences (*p* < 0.05); values with the same or no superscript letters indicate no significant difference (*p* > 0.05).

**Table 5:** Effect of glycerol fatty acid esters on Serum biochemical Indices and Antioxidant Indices of Dabieshan Beef Cattle.

Items	CON	0.05GFAE	0.1GFAE	SEM	*p*-Value
TP (g/L)	65.56 ^b^	69.78 ^ab^	74.11 ^a^	1.28	0.010
ALB (g/L)	26.76	25.92	26.77	0.98	0.931
UREA (mmol/L)	5.16 ^a^	3.99 ^b^	3.43 ^b^	0.24	0.002
TG (mmol/L)	0.12	0.16	0.16	0.02	0.781
TC (mmol/L)	3.82	3.01	2.09	0.34	0.113
LDL-C (mmol/L)	0.34	0.28	0.28	0.03	0.594
HDL-C (mmol/L)	1.46	1.32	1.42	0.06	0.558
T-AOC (μmol/mL)	1.06 ^c^	1.42 ^b^	1.55 ^a^	0.05	0.001
SOD (nmol/mL)	170.40 ^b^	182.84 ^a^	184.01 ^a^	2.37	0.020
MDA (nmol/mL)	8.74 ^a^	6.97 ^ab^	6.29 ^b^	0.44	0.040
GSH-PX (nmol/mL)	119.98	124.08	126.84	2.60	0.591
CAT (nmol/mL)	11.3	11.61	10.45	5.44	0.950

CON: basal diet; 0.05 GFAE: basal + 0.05% GFAE of dietary dry matter; 0.1 GFAE: basal + 0.1% GFAE of dietary dry matter. ALB: albumin; TP: total protein; TG: triglycerides; UREA: urea; LDL-C: low-density lipoprotein cholesterol; TC: total cholesterol; SOD: superoxide dismutase; HDL-C: high-density lipoprotein cholesterol; T-AOC: total antioxidant capacity; MDA: malondialdehyde; CAT: catalase; GSH-PX: glutathione peroxidase. SEM: Standard Error of the Mean; Distinct superscript letters (a, b, c) in the same row denote statistically significant intergroup differences (*p* < 0.05); values with the same or no superscript letters indicate no significant difference (*p* > 0.05).

**Table 6:** Glycerol fatty acid esters on volatile fatty acids in the rumen of Dabieshan beef cattle.

Items	CON	0.05GFAE	0.1GFAE	SEM	*p*-Value
pH	6.75	6.63	6.65	0.05	0.085
NH_3_-N (mg/100 mL)	14.01	12.73	13.11	0.65	0.210
Acetate (mmol/L)	54.18	55.74	58.67	1.62	0.579
Propionate (mmol/L)	13.66	13.74	17.15	0.87	0.197
Butyrate (mmol/L)	4.66 ^b^	5.47 ^a^	5.50 ^a^	0.27	0.025
Isobutyric acid (mmol/L)	0.74	0.77	0.88	0.14	0.271
Isovaleric acid (mmol/L)	0.81	0.83	0.92	0.22	0.811
Valeric acid (mmol/L)	0.48	0.64	0.52	0.03	0.271
TVFA (mmol/L)	74.55	77.26	83.66	2.23	0.599
A/P	3.99	4.13	3.47	0.14	0.125

CON: basal diet; 0.05 GFAE: basal + 0.05% GFAE of dietary dry matter; 0.1 GFAE: basal + 0.1% GFAE of dietary dry matter; NH_3_-N:ammonia-nitrogen; TVFA: Total volatile fatty acid; A/P: acetate/propionateratio. SEM: Standard Error of the Mean; Distinct superscript letters (a, b) in the same row denote statistically significant intergroup differences (*p* < 0.05); values with the same or no superscript letters indicate no significant difference (*p* > 0.05).

**Table 7:** Glycerol fatty acid esters on Methane emission and Carbon Dioxide Emissions of Dabieshan beef cattle.

Items	CON	0.05 GFAE	0.1 GFAE	SEM	*p*-Value
CH_4_ (g/d)	147.71 ^a^	137.51 ^b^	130.54 ^b^	2.16	0.005
CO_2_ (g/d)	3264.48	3336.55	3405.86	79.96	0.796

CON: basal diet; 0.05 GFAE: basal + 0.05% GFAE of dietary dry matter; 0.1 GFAE: basal + 0.1% GFAE of dietary dry matter; CH_4_: Methane; CO_2_: Carbon Dioxide. SEM: Standard Error of the Mean; Distinct superscript letters (a, b) in the same row denote statistically significant intergroup differences (*p* < 0.05); values with the same or no superscript letters indicate no significant difference (*p* > 0.05).

## Data Availability

The original contributions presented in this study are included in the article/Supplementary Material. Further inquiries can be directed to the corresponding authors.

## Supplementary Materials

The following supporting information can be downloaded at: https://www.mdpi.com/article/10.3390/vetsci13010092/s1, Supplementary Method S1: DNA Extraction and Sequencing.

## Abbreviations

This manuscript employs the following abbreviations in subsequent content: IBW, initial body weight; FBW, final body weight; TBW, total body weight gain; ADG, average daily gain; DMI, dry matter intake; GFAE, glycerol fatty acid esters; CH_4_, methane; CO_2_, carbon dioxide; CON, control; L*, lightness; a*, red-green chromaticity coordinate; b*, blue-yellow chromaticity coordinate; EMA, eye muscle area; CP, crude protein; EE, ether extract; TP, total protein; ALB, albumin; UREA, urea nitrogen; TC, total cholesterol; TG, triglycerides; HDL-C, high-density lipoprotein cholesterol; LDL-C, low-density lipoprotein cholesterol; T-AOC, total antioxidant capacity; SOD, superoxide dismutase; MDA, malondialdehyde; GPX, glutathione peroxidase; CAT, catalase; TVFA, total volatile fatty acid; A/P, acetate/propionate ratio.

## Appendix A

**Table A1:** Effects of glycerol fatty acid esters at the species level in the rumen of Dabieshan beef cattle.

Items	CON	0.05 GFAE	0.1 GFAE	SEM	*p*-Value
*Bacteroidales*_bacterium	38.76	30.79	39.38	1.792	0.101
*Prevotella*_sp.	11.93 ^b^	19.15 ^a^	16.52 ^a^	0.969	0.001
*Bacteroidaceae*_bacterium	6.978	6.22	8.23	0.641	0.478
*Clostridia*_bacterium	6.476	4.59	4.47	0.732	0.490
*Paludibacteraceae*_bacterium	3.10	1.62	2.75	0.515	0.532
*Oscillospiraceae*_bacterium	1.69	1.92	1.38	0.145	0.378

CON: basal diet; 0.05 GFAE: basal + 0.05% GFAE of dietary dry matter; 0.1 GFAE: basal + 0.1% GFAE of dietary dry matter; SEM: Standard Error of the Mean; Distinct superscript letters (a, b) in the same row denote statistically significant intergroup differences (*p* < 0.05); values with the same or no superscript letters indicate no significant difference (*p* > 0.05).
