# Supplementary material for: Effects of Glycerol Fatty Acid Esters on Growth Performance, Methane Emissions, and Rumen Microbial Flora of Dabieshan Beef Cattle
Source: Vet Sci. 2026 Jan 16;13(1):92. doi: 10.3390/vetsci13010092 (PMC12846351; doi:10.3390/vetsci13010092)
Supplement: Supplementary file 1 [file vetsci-13-00092-s001.zip › vetsci-4068319-supplementary.pdf]

Supplementary Method S1

DNA Extraction and Sequencing

DNA from the genome was extracted employing the Kit, followed by an assessment of both concentration and purity. To evaluate the integrity of the isolated DNA, 1% agarose gelelectrophoresis was performed. Next, fragmentation of the DNA was carried out using the Covaris M220 device, selecting fragments of around 350 bp for the construction of paired-end (PE) sequencing libraries. During library preparation, "Y"-shaped adapters were initially ligated to the DNA fragments, followed by magnetic bead purification to eliminate self-ligated adapter fragments. The library template was amplified via PCR, and DNA was denatured with NaOH to produce single-stranded fragments. In the bridge PCR step, one end of the DNA fragment was hybridized and immobilized to the primer on the flow cell, while the other end randomly hybridized with a nearby primer and was also immobilized, forming a "bridge" structure. Subsequent PCR amplification generated DNA clusters, which were linearized into single-stranded DNA. Metagenomic sequencing was subsequently performed on the Illumina NovaSeq platform by General biol Co., Ltd. (Anhui, China). During sequencing, modified DNA polymerase and four types of fluorescently labeled dNTPs were added, with only one base incorporated per cycle. The reaction surface was scanned with a laser to detect the nucleotide type incorporated in the first round of each template sequence. The "fluorescent group" and "terminator group" were chemically cleaved to restore the 3' end, allowing the polymerization of the next nucleotide. The sequence of the template DNA fragment was determined by analyzing the fluorescent signals collected in each cycle.

Sequencing Data Processing

To analyze the sequencing data obtained in this study, the following workflow was

implemented: Fastp v0.20.0 was employed for quality control of reads containing index tags, removing low-quality sequences and host DNA contamination. Subsequently, MEGAHIT v1.1.2 was used to perform de novo assembly of the quality-controlled reads, selecting contigs of at least 300 bp in length. Prodigal v2.6.3 was then utilized to predict open reading frames (ORFs), and genes with a nucleotide length of at least 100 bp were selected and translated into amino acid sequences. To construct a non-redundant gene set, CD-HIT v4.6.1 was used to cluster the predicted gene sequences (sequence identity ≥ 90%, coverage ≥ 90%). SOAPaligner v2.21 was subsequently employed to align the high-quality reads to the non-redundant gene set for gene abundance quantification. Finally, DIAMOND v0.8.35 was used to perform BLASTP alignment of the amino acid sequences of the non-redundant gene set against the NR database (E-value ≤ 1e-5) to obtain species-level annotations of the genes. Species abundance was then calculated, and abundance profiles were constructed at various taxonomic levels for microbial community structure analysis.
